# The dynamic interaction between COVID-19 and shipping freight rates: a quantile on quantile analysis

**DOI:** 10.1186/s12544-022-00566-x

**Published:** 2022-09-16

**Authors:** Khalid Khan, Chi Wei Su, Adnan Khurshid, Muhammad Umar

**Affiliations:** 1 School of Finance, Qilu University of Technology, Jinan, China; 2 School of Economics, Qingdao University, Qingdao, China; 3 School of Economics and Management, Zhejiang Normal University, Jinhua, China

**Keywords:** Shipping industry, Freight rate, Coronavirus, Quantile-on-quantile, Wavelet transforms, F10, G01, J30, R15

## Abstract

This study determines the impact of the coronavirus disease (COVID-19) that has been prevalent since the year 2019, on the shipping freights. This task has been undertaken by using the wavelet quantile on the quantile approach. The results of the study affirm that the pandemic has in fact affected the shipping freight costs, primarily due to the lower demand for energy and raw materials, and the unavailability of the vessels. In addition to this, the spread of COVID-19 has had a positive impact on the Baltic Dry Index in the high quantiles and is deemed to be more responsive in the long run. Also, the COVID-19 infection has had a negative effect on the Baltic Dry Tanker Index and the Baltic Clean Tanker Index in the medium to high quantiles, particularly in the short and the medium run. The positive impact of COVID-19 on the Baltic Clean Tanker Index has been recognized in the long term in the high quantiles. These findings support the theoretical model which states that the spread of COVID-19 and the shipping freights are closely related. The results suggest that the degree of the effect is more causal in the short. Therefore, the shipping industry must ideally pay special attention to the detection of abrupt changes in the freight rate dynamics, and the specific regulations regarding these intricacies are critical.

## Introduction

The shipping industry is considered to be the main pillar of international trade, and about 80% of the goods around the globe are believed to be transported through sea [13, 43]. However, the uncertainty caused by geopolitical, economic and pandemic-related factors may disrupt the supply of goods, and therefore exert severe consequences on maritime trade [14]. In this regard, the pandemic has prompted a global socioeconomic crisis, and mobility restrictions have been adopted to control the spread of the virus [24]. In addition to this, it has created an economic emergency that has far-reaching consequences for marine transportation, ports, and shipping [10]. Also, as a result of the pandemic, freight costs have fluctuated aggressively, thus owing to sluggish economic development and demand for raw materials. As the shipping demand is linked to the economy, the spread of the COVID-19 pandemic has adversely affected the economy, a change that has been reflected in the fluctuating freight prices [26]. The demand shock caused by COVID-19 has disrupted the global economy, thus resulting in a fall in demand for transportation services [27]. Moreover, it can also be affirmed with evidence that the industry has been hit hard by the pandemic, as the maritime trade has slipped drastically due to the supply disruption that has taken place as a result of material shortages, port closures and labor unavailability [43]. Meanwhile, the freight costs have touched the highest levels, a development that has led to considerable consequences for commodity prices and inflation. Therefore, the study pertaining to shipping freight rate response to the COVID-19 is of utmost importance.

The spread of the COVID-19 virus is regarded as the most serious economic catastrophe, and it is considered to be even more grave than the subprime mortgage crisis [30, 38]. The global lockdown restrictions in the first quarter of 2020 essentially resulted in the highest levels of uncertainty, causing the demand for commodities, raw materials and energy to fall, thus resulting in the lowest levels of freight cost [38, 45]. In addition to this, the strict lockdowns and the production closure in Europe and the U.S. have impeded marine trade; a development that has led to a lower demand for shipping as a mode of transport and trade. However, due to a slight economic rebound experienced in numerous Asian nations, the maritime sector started showing signs of life in the second quarter of the year 2020. Meanwhile, trade flows from China to Europe and the U.S. were observed to be growing, but not enough ships were available to keep up with the demand, thus resulting in rising freight rates. However, demand was observed to have gradually recovered in the year 2021, and supply also showed signs of improvement, while the energy prices led to an increase in the demand for big crude oil tankers. Then, In the second quarter of the year 2021, raw material demand increased, resulting in the highest amount of shipping freight ever to have been recorded. Moving on, the freight costs experienced a fall in October 2021, due to the stability of the supply chain. The current highs in the freight prices in this time period were observed to be mostly due to the pandemic-related shocks, and the unanticipated spikes in the shipping demands [42]. Meanwhile, the trade was seen to have climbed above the pre-COVID-19 era level. This was primarily because of the surge in economic recovery, thus putting pressure on freight costs. As a result, the volatility factor related to the shipping freight indices, such as the Baltic Dry Bulk Index (BDI), Baltic Dirty Index (BDTI), and the Baltic Clean Tanker Index (BCTI), as induced by the pandemic must be taken into a close and critical evaluation, and the policy implications might be a helpful input for the relevant stakeholders.

This study adds to the existing literature in various ways. In the first instance, it evaluates the influence of the COVID-19 on major international shipping freights. It is common knowledge that the majority of global trade is handled by sea, and the shocks produced by the COVID-19 uncertainty have had a significant impact on freight costs. Fortunately, the highest freight level has been observed due to the pandemic, which has a drastic effect on the commodity prices, as weak as inflation. Meanwhile, the higher cost of shipping was deemed to be a severe setback to the economic recovery in the post-pandemic period. So much so that it renders a macro correlation and induces influence across various quantiles. It can be observed that the outcomes display that the pandemic has affected shipping freight in the upper quantile. Similarly, it is a part of the research to observe and analyze the impact of COVID-19 on key maritime freights such as BDI, BDTI, and BCTI. This adds to the current literature that has been using different analysis approach. Moreover, the study undertakes the impact of COVID-19 on freight costs in three different modes which include the full sample, short, medium and long-run periods. Lastly, the paper contributes to the present literature in terms of its econometric methodology – a phenomenon that combines the wavelet transform, and the quantile-on-quantile (QQ) procedure. Therefore, it may be the first attempt to use the methodology for the examination of the impact of COVID-19 on the shipping freight costs, which provide a useful contribution. The technique is valuable to study the nonlinear association between the series in various quantiles.

The results suggest that the pandemic has severely affected the shipping freight costs primarily due to the lower demand for raw materials, energy, unavailability of the vessels, increase in the distances of voyages, and logistical inefficiencies. In the high quantiles, the spread of COVID-19 has had a continuously positive influence on the freight prices, thus showing that the COVID-19 induced uncertainty has led to an even more elevation of these costs. However, it has also been observed that the freight cost is more responsive to the supply disruption and rising demand created by the COVID-19, particularly in the long term. The outcomes are in line with the theoretical model, which essentially states that the COVID-19 infection and shipping freight are closely correlated. Therefore, it is imperative that the shipping industry pays heed towards detecting such sudden changes in the freight rate dynamics and regulations. Moreover, the global economy has a derivative effect on the industry, and the uncertainty can be disastrous to the commodity prices, as well as future inflationary pressures.

The rest of the paper has been structured in the following manner: It highlights the literature review in Sect. 2, while the quantile-on-quantile method has been described in Sect. 3. This is followed by the theoretical model in Sect. 4, while the data trends and summary are described in Sect. 5. Moving on, the empirical analysis of the impact of COVID-19 on the shipping costs by the quantile-on-quantile approach has been explained in Sect. 6. This study is then concluded, and the policy implications that are recommended have been presented in Sect. 7.

## Literature review

The extant literature consists of studies that determine the pandemic’s impact on the logistical operations. In this regard, Yazir et al. [46] explore that the pandemic has shaken the global economy, and has had a considerable impact on the shipping industry due to the falling demand for goods. In this regard, According to Rewari et al. [34], different enterprises and trade channels may be connected by developing activities that are efficient for carrying out smooth trade across countries and trade routes. At another instance, a study by Thuy et al. [39] revealed that businesses may leverage various modes of transportation in order to develop expansion possibilities and implement initiatives to improve trade and portability in their countries. Also, Gray [5] confirmed that often, utilized transportation routes are employed by companies to deal with supply chain operations. Klatman et al. [17] show that the ports and border restrictions caused by COVID-19 essentially made it difficult for firms to deliver materials that are to be transported through sea. In addition to this, Kwon [19] concluded that global shipping is a mode of freight transfer that has been severely impacted through the sea routes in terms of carrying commodities to other countries. Ivanov & Das [11] also found that health issues, as a result of COVID-19, caused a decline in the number of crew workers at the seaports. Gu et al. [6] took this opportunity to show that BDI has a better hedging performance as compared to the other freights. Moreover, Xu et al. [44] examined the Chinese transportation and logistics behavior during the peak times of the COVID-19 pandemic. The results of these studies essentially suggest that the pandemic does not affect the maritime costs.

Some of the studies have shown the impact of COVID-19 on different shipping freights. One such study is by Arifin [1], who evaluates the nexus between international shipping freight and COVID-19. Results of the study confirm that the pandemic has in fact squeezed the demand for goods, which has had a negative effect on the international freight costs. In this regard, Michail and Melas [26] have found that the pandemic has reduced the demand for goods, primarily due to the restrictions that have been levied to contain the virus which has significant consequences on the BDI and BDTI. Here, Khan et al. [13] assess the impact of oil price and geopolitical risks, on the BDI. They have concluded that the oil price fluctuations have considerable consequences for BDI in the short run. Moreover, the magnitude of the effect is more visible and stronger during times of higher geopolitical risks. Khan et al. [14] also evaluated the BDTI response to global economic uncertainty and oil price volatility, and the outcomes confirm that the BDTI is extremely vulnerable to oil price fluctuations. At another instance, Menhat et al. [25] reported that the COVID-19 pandemic has had a negative influence on the Malaysian shipping industry as well. Furthermore, Millefiori et al. [28] suggested that a lockdown, paired with a border closure has dramatically declined the maritime mobility, a phenomenon which has ultimately been reflected in the shipping costs. Moving on, Tianming et al. [40] have also inspected the impact of COVID-19 on the ocean trade and supply chains. The results have confirmed that COVID-19 has caused strict lockdowns and squeezed the economic activity, maritime transport and freight rate. Also, Notteboom et al. [31] have considered the supply shocks of the COVID-19 effect on the shipping industry and container ports. The findings suggest that the pandemic has affected the supply chains and port activity, particularly when it comes to vessel calls and container volumes which translates into shipping freight.

In a similar study, Jacks and Stuermer [12] have found that shipping demand shocks have more of an impact as compared to the fuel prices and supply shocks. In this regard, Gu et al*.* [7] have found that the fuel and commodity prices and the financial markets are considered to be the main determinates of the shipping market. Also, Mańkowska et al. [23] have shown that the spread of COVID-19 has differently impacted maritime supply chains. Moreover, some of them are completely closed, while some have the lowest operations and vice versa. In addition to this, Łącka et al. [20] have also investigated the pandemic’s effect on Poland’s shipping industry and have determined that the pandemic has adversely affected freight costs. In this context, Oyenuga [32] also finds that COVID-19 has led to a decline in the maritime trade, re-routed shipments, and faced bankruptcies in the short run, which has had adverse consequences on the shipping cost.

## Theoretical model

The modified Beenstoke [2] model has been used to analyze the nexus between COVID-19 and shipping freight. the model revolves around the idea of a highly competitive freight market, which contains many factors, such as oil prices, global trade volume, and shipping service demand that may potentially affect transportation costs. However, the emergence of COVID-19 has squeezed the economic growth, wherein the oil prices have collapsed and there has been a decline in the shipping demand. Thus, shipping demand can be illustrated as follows.

1 $$DS_{t} = f\left( {\frac{{GMT_{t} + FC_{t} }}{{COVID_{t} }}} \right)$$

Here, $DS$ represents the shipping service demand, Covid denotes the factor for coronavirus, GMT denotes the global maritime trade, and *FC* denotes the freight cost. It also shows that the demand for shipping services is related to the global maritime trade, which may eventually elevate the shipping costs. Therefore, rearranging Eq. (1) for FC suggests that global trade and shipping demand are the leading factors of the freight rate.

Equation (2) explains that the trade increases may cause a push in the demand for shipping which then has to incur oil costs to ship goods from the producer to the consumer and pushes the freight costs. This relationship happens to be more pronounced if there are uncertainties that can increase the disruption of supply. Hence, in this way, Eq. (2) for *FC* as a function of demand for shipping services, global maritime trade, and COVID-19 is estimated as follows:

2 $$FC_{t} = \phi_{0} + \phi_{1} COVID - 19_{t} + \phi_{2} GMT_{t} + \phi_{3} DS_{t} + \varepsilon_{t}$$

It demonstrates that COVID-19 has harmed the global economy, as seen by the increased shipping freight costs. It is noteworthy that COVID-19 has severe economic ramifications for marine transportation, ports, and shipping [10]. Moreover, the lockdown and mobility restrictions have created the greatest degree of uncertainty, causing the demand for commodities, raw materials, and energy to fall, thus resulting in the lowest level of freight cost and vice versa.

## Methodology

### Wavelet analysis

The wavelet QQ technique has been utilized to address the shortcomings of the prior approaches. The approach may be used to evaluate any sample size, regardless of whether the series is dyadic in nature or not [9]. In a similar manner, the multivariate analysis may also be handled using the wavelet QQ technique [3, 16]. this technique can essentially enhance the capacity to detect dependency in the entire sample [8]. As a result, the preceding approaches' potential to ignore the nature of changes, including large and small changes, can have a significant impact on a relationship [4]. Moreover, the variables are affected differently by the positive and negative shocks. Also, the asymmetric association is not considered in the investigation. However, the QQ technique successfully evaluates the dependency as a whole and offers a time-varying effect at each point [8]. To put it briefly, the QQ technique provides a level of flexibility that identifies the functional dependence relationship between the series [16].

In economics, the wavelet approach is widely used, and the wave fluctuation starts at zero and returns to zero [36, 37, 47]. The wavelet with various frequencies is adequate for both the time and frequency sphere [41]. Therefore, the wavelet is made dyadically and alters the functions like $\rho$ and $\sigma$:

3 $$\smallint \rho \left( t \right)dt = 1$$

4 $$\smallint \sigma \left( t \right)dt = 0$$

where $\rho$ and $\sigma$ mean the basic wavelet, respectively. The previous notices the even and low-frequency modules of the series, and later identifies the inclusive and upper-frequency modules of the series. So, the realized wavelet has been demonstrated as follows.

5 $$\rho_{u,v} \left( t \right) = 2^{\frac{u}{2}} \rho \left( {2^{u} t - v} \right)$$

6 $$\sigma_{u,v} \left( t \right) = 2^{\frac{u}{2}} \sigma \left( {2^{u} t - v} \right)$$

Moreover, the number of clarifications controls the maximum number of scales that the research can estimate (*T* ≥ $2^{u}$).

A distinct aspect of the wavelet extension is the coefficient of the setting feature $\rho_{u,v} \left( t \right)$, which connotes the evidence of the function at the assessed setting $v2^{ - u}$, and frequency $2^{u}$. Therefore, the $L^{2} \left( {\mathbb{R}} \right)$ can be a lengthened fundamental wavelet at the random point $u_{0} \in {\mathbb{N}}$, over diverse scales.

7 $$X\left( t \right) = \mathop \sum \limits_{q} Q_{uo, v} \rho_{uo,v} \left( t \right) + \mathop \sum \limits_{u > uo} \mathop \sum \limits_{v} d_{u,v} \rho_{u,v} \left( X \right) \ldots \ldots \ldots \ldots u = u_{0} \ldots \ldots .u$$

where the function $\rho_{uo,v}$ symbolizes a scaling function, and the equivalent uneven scale coefficients $Q_{uo, v}$ and $d_{u,v}$ represent the inclusive coefficients stated by $Q_{uo, v} = \smallint X\left( t \right)\rho_{u,v} \left( t \right)dt$ and $d_{u,v} = \smallint X\left( t \right)\sigma \left( t \right)dt$, respectively. Moving on, the series $Q_{u,t} = \mathop \sum \limits_{v} Q_{u0,v} \rho_{vo,u}$(t) deals with a smooth system of the primary variable (*t*), which identifies the long-term (i.e., low-frequency) aspects. However, the series $D_{u,t} = \mathop \sum \limits_{v} d_{u,v} \sigma_{u,v}$(t) detect the local deviations (i.e., the higher-frequency attributes) of (*t*).

### Maximum overlap discrete wavelet transforms

The maximum overlap discrete information transform is achieved through the “discrete sampled by discrete wavelet transforms (DWT). This particular transform is subject to the scaling filter ($s_{l}$, *l* = 0……. *L* − .$1^{2}$), and the wavelet filter ($f_{l} ,l = 0, \ldots .,L - 1^{3} )$. in this regard, the *L* ∈ ℕ indicates the length of the filter [33]”. Therefore, the wavelet filter justifies these three features.

8 $$\mathop \sum \limits_{l - 0}^{L - 1} s_{l} = 0, \mathop \sum \limits_{l - 0}^{L - 1} s_{l}^{2} = 0, \mathop \sum \limits_{l - 0}^{L - 1} s_{l} s_{l + 2n} = 0\forall {\text{ n}} \in {\mathbb{N}}$$

The small and upper-pass filters are expounding as the quadrature mirror filters,

9 $$s_{l} = \left( { - 1} \right)^{l} s_{L - 1 - l} {\text{or }}s_{l} = \left( { - 1} \right)^{l + 1} s_{L - 1 - l} = l = 0 \ldots .., L - 1$$

Also, the scaling filter justifies the settings.

10 $$\mathop \sum \limits_{l = 0}^{L - 1} f_{l} = \sqrt 2 \mathop \sum \limits_{l = 0}^{L - 1} f_{l}^{2} = 1 {\text{and}} \mathop \sum \limits_{l = 0}^{L - 1} f_{l} f_{l + 2n} = 0 \forall {\text{ n}} \in {\mathbb{N}}{ }$$

Moreover, the DWT scaling coefficients at the *u*th level for *u* ∈ {1,…, *u*} are expounded as:

11 $$p_{{{\varvec{u}},\user2{ t}}} = \mathop \sum \limits_{l = 0}^{L - 1} s_{l} X_{t - 1} {\text{and}} q_{{{\varvec{u}},\user2{ t}}} = \mathop \sum \limits_{l = 0}^{L - 1} f_{l} X_{t - 1}$$

For this purpose, Percival and Walden [33] recommend the use of the maximal intersection discrete wavelet transform (MODWT) to divide the series. This technique is valuable in resolving the DWT restrictions. Moreover, the Daubechies minimum asymmetry and the scaling factor realize the wavelet because of its powerful ability to detect a series of time scale deviations.

The primary series has been divided into different frequency bands. Therefore, the modified scaling is reached by integrating the MODWT as follows.

12 $$\tilde{s}_{p, l} = \frac{{s_{u, l} }}{{2^{\frac{u}{2}} }} \; {\text{and}}\; f_{u, l} = \frac{{f_{u, l} }}{{2^{u/2} }},\quad u = 0, \ldots , u{ }$$

As endorsed and affirmed by Mallat [22], $\tilde{s}_{u,t}$ and $\tilde{f}_{u,t}$ are realized by employing "the pyramid algorithm, which needs three responses for each duplication of the MODWT algorithm. The first one of these begins by vacillating data and offers the scaling coefficients and wavelet”.

13 $$\tilde{s}_{1,t} \mathop \sum \limits_{l - 0}^{L - 1} \tilde{s}_{l} X_{t - 1} \;and\; \tilde{f}_{1,t} = \mathop \sum \limits_{l - 0}^{L - 1} \tilde{f}_{l} X_{t - 1}$$

The scaling factor of the first step has been developed as the input data vector, to accomplish the second phase”. The second level wavelet is demonstrated below.

14 $$\tilde{s}_{2, t} = \mathop \sum \limits_{l - 0}^{L - 1} \tilde{s}_{l} \tilde{f}_{1, t - l } {\text{mod }}\;{\text{N}}\;{\text{and}}\;{ }\tilde{f}_{2, t} = \mathop \sum \limits_{l - 0}^{L - 1} \tilde{f}_{l} X_{t - l} \;{\text{ mod}}\;{\text{ N }}$$

Also, the *u*th level MODWT wavelet and scaling coefficients of the time series *Xt* are stated as:

15 $$\tilde{s}_{u, t} = \mathop \sum \limits_{l - 0}^{L - 1} \tilde{s}_{l} \tilde{f}_{1, t - l } {\text{mod N and }}\tilde{f}_{u, t} = \mathop \sum \limits_{l - 0}^{L - 1} \tilde{f}_{l} X_{t - l} {\text{mod N }}$$

### The quantile-on-quantile method

The quantile regression does not deliberate the nature of large and small fluctuations that influence the relationship [4]. The asymmetric association, like the positive shock, has a different effect as compared to the negative shock which is not evaluated. Therefore, the QQ technique is used to distinguish the reliance in its total, so that the association between variables could fluctuate at each point of their respective distributions. Similarly, the method provides a comprehensive picture of reliance [8]. It can explore the influence of shocks at changeable extent and heterogonous tail dependence. The procedure has the benefit of elasticity, which perceives the functional arrangement of the link between series”.

Therefore, this study concisely elucidates the attribute of the QQ technique as recommended by Sim and Zhou [35], in order to probe the COVID-19 effect on shipping freight. The technique is employed to spot the influence of COVID-19 on shipping freight. Thus, this study has utilized the QQ methodology to analyze the influence of the quantiles of COVID-19 on the different shipping freight such as BDI, BDTI and BCTI.

In order to apply the technique, The nonparametric quantile regression model is the initial step.

16 $${\text{FC}} = {\upgamma }^{\varphi } \left( {{\text{COVID}} - 19_{t} } \right) + \varepsilon_{t}^{\varphi }$$

where FC is the freight rates such as BDI, BDTI and BCTI.

The study analyzes the relationship between the ${{\upvarphi }}$ th quantiles in the context of COVID-19^τ^ through the local linear regression [15]. As ${\upgamma }^{\varphi } \left( . \right)$ is a nameless function, “this function can be assessed by a first-order Taylor expansion about the quantile COVID-19^τ^”. Therefore,

17 $${\upgamma }^{\varphi } \left( {{\text{COVID}} - 19_{t - 1} } \right) = {{\upgamma^{\prime\prime}}}\left( {{\text{COVID}} - 19{ }^{\tau } } \right) + {\upgamma }^{\varphi } \left( {{\text{COVID}} - 19{ }^{\tau } } \right) \left( {{\text{COVID}} - 19{ }_{t - 1} - COVID^{\tau } } \right)$$

where ${\upgamma }^{\varphi }$ displays the partial derivative of ${\upgamma }^{\varphi } \left( {{\text{COVID}}_{t - 1} } \right)$ in the background of COVID-19. The result is named the marginal effect and accepts the slope coefficient in the linear regression model.

The limitations ${\upgamma }^{\varphi } \left( {{\text{COVID}} - 19_{{{\text{t}} - 1}} } \right)$ and $\beta^{\varphi } \left( {{\text{COVID}} - 19^{{\uptau }} } \right)$ are a visible feature of Eq. (18) that are twice as indexed in ${{\upvarphi }}$ and τ. Supposed that ${\upgamma }^{\varphi } \left( {{\text{COVID}} - 19_{{{\text{t}} - 1}} } \right)$ and ${\upgamma }^{\varphi } \left( {{\text{COVID}} - 19^{{\uptau }} } \right)$ are both functions of ${{\upvarphi }}$ and ${\text{COVID}} - 19^{{\uptau }}$, and that ${\text{COVID}} - 19^{{\uptau }}$ is a function of τ, it is clear that both ${{ \upgamma }}^{\varphi } \left( {{\text{COVID}} - 19_{{{\text{t}} - 1}} } \right)$ and ${\upgamma }^{\varphi } \left( {{\text{COVID}} - 19^{{\uptau }} } \right)$ are functions of θ and τ. Moreover, the functions ${\upgamma }^{\varphi } \left( {{\text{COVID}} - 19_{{{\text{t}} - 1}} } \right)$ and $\gamma^{\varphi } \left( {{\text{COVID}} - 19^{{\uptau }} } \right)$ can be retitled as ${\upgamma }\left( {{{\upvarphi }},{{ \uptau }}} \right)$ and ${\upgamma }_{1} \left( {{{\upvarphi }},{{ \uptau }}} \right)$, respectively. Thus, Eq. (18) can be restated as:

18 $$\gamma^{\varphi } \left( {{\text{COVID}} - 19{ }_{t - 1} } \right) = \gamma^{\varphi } \left( {\varphi , \tau } \right) + \gamma_{1} \left( {\varphi , \tau } \right)\left( {{\text{COVID}} - 19_{t - 1} - {\text{COVID}} - 19^{\tau } } \right)$$

By substituting Eq. (13) into (15), and extracting (17):

19 $$\underbrace {{FC_{t} = \gamma_{0} \left( {\varphi , \tau } \right) + \gamma_{1} \left( {\varphi , \tau } \right)\left( {{\text{COVID}} - 19_{t - 1} - {\text{COVID}} - 19^{\tau } } \right){ }}}_{*} + \varepsilon_{t}^{{{\upvarphi }}}$$

where FC denotes the BDI, BDTI and BCTI. The part ( ∗) of Eq. (19) denotes the $\varphi$ th conditional quantiles of COVID-19. But, distinct from the function of the regular conditional quantiles, this expression repeats the relationship between the $\varphi$ th quantiles of *COV*ID, and the τth quantiles of shipping freight, due to the limitations $\gamma_{0}$ and ${\upgamma }_{1}$, which are doubly indexed in ${\upgamma }$ and τ. Similarly, a linear relation is not assumed at any time between the quantiles of the series [15].

It can then assess that Eq. (20) needs the substituting of ${\text{COVID}} - 19_{t - 1}$ and ${\text{COVID}} - 19^{\tau }$ with their expected peer ${\text{COVID}} - 19_{t - 1}$ and ${\text{COVID}} - 19^{\tau }$, respectively”. The local linear regression evaluations of the limits $\sigma_{0}$ and $\sigma_{1}$, which are the evaluations of ${\upgamma }_{0}$ and ${\upgamma }_{1}$, respectively, have been achieved by explaining the minimization problem:

20 $$\mathop {\min }\limits_{{\sigma_{0} ,{ }\sigma_{1} }} \mathop \sum \limits_{i = 1}^{n} \rho_{{{\upvarphi }}} [FC_{t} - \sigma_{0} - \sigma_{1} (\widetilde{{{\text{COVID}} - 19_{t - 1} }} - \widetilde{{{\text{COVID}} - 19^{\tau } }})] \times K\left( {\frac{{F_{n} \widetilde{{\left( {{\text{COVID}} - 19{\uptau }} \right) - {\uptau }}}}}{h}} \right)$$

where $\rho \varphi \left( \varepsilon \right)$ is the quantile loss function, explained as $\rho \varphi \left( \varepsilon \right) = \varepsilon \left( {\varphi - {\text{I}}\left( {{\upvarepsilon } < 0} \right)} \right)$ and *I* specifies the normal display function. Then, “*K* (∙) represents the Gaussian kernel function and ℎ represents the bandwidth parameter of the kernel. It determines the size of the neighborhoods about the target point and explores the smoothness of the resulting estimates. Hence, the selection of the bandwidth is more important in the nonparametric estimation method. The estimation can cause biased results when the large bandwidth is selected, and a higher variance with the smaller bandwidth. Thus, the appropriate bandwidth selection is critical to provide a balance between the bias and the variance. Moreover, the constant bandwidth is not appropriate for every situation and may result in a factor of biases in estimation [21]. This study, therefore, has employed the bandwidth parameter ℎ = 0.05 for the estimation based on Sim and Zhou [35] methodological method.”

#### Data

The COVID-19 effect on the shipping freight rates such as BDI, BDTI and BCTI have been measured from a time period pertaining to 2020/01/20 to 2022/02/28. This period is critical due to the frequent changes in the shipping freights because of the breakout of COVID-19. The global situation has changed thus far, and the maritime trade is being restricted, affecting the international shipping business by a significant measure. Moreover, the global economy is being disrupted, and industrial operations are curtailed, which has an impact on the supply and logistics [43]. Also, the cost of shipping products from Asia to Europe and the U.S. has reached an all-time high, owing to the shortage of containers during the pandemic. it can also be observed that the COVID-19 data consists of newly infected cases on a daily basis- an information that is obtained from the World Health Organization (WHO). Similarly, the freight costs are shown by BDI, BDTI and BCTI which are retrieved from the Baltic Exchange London. It must be noted that the average dry bulk material transportation price is referred to as the BDI, which is used to describe the economic forecast. However, BDTI is the average worldwide oil shipment cost for the 12 international routes. It can be affirmed that the BCTI tracks the costs of tankers that transport the cleansed cargo of oil products such as gasoline, diesel and heating oil. The summary statistics have been illustrated in Table 1. It reveals that the BDI has the highest standard deviation in the shipping freights, which shows the frequent fluctuations that have been taking place during the pandemic. On the other hand, COVID-19 cases have shown a higher standard deviation as confirmed by the different phases of the pandemic, and therefore, this leads to a higher level of uncertainty. The skewness values indicate that BDI and COVID-19 have negative values, while BDTI and BCTI are skewed positively. Moreover, the leptokurtic distribution has been detected except for BDI through the kurtosis values, as the values exceed a level of 3. In this regard, The Jarque–Bera test confirms the non-normally distribution of all the series.

**Table 1:** Summary statistics

	BDI	BDTI	BCTI	COVID-19
Mean	7.393	6.438	6.235	17.172
SD	0.684	0.302	0.319	2.558
Skewness	− 0.335	0.914	1.653	− 1.739
Kurtosis	2.220	3.593	7.727	5.747
Jarque–Bera	20.614^***^	72.097^***^	648.712^***^	382.956^***^

***1% significance level.

The shipping freights and COVID-19 correlation has been illustrated in Table 2. The outcomes explore the correlation of BDI with the pandemic, followed by BDTI and BCTI. At a 1% significance level, the association that has been observed is critically significant.

**Table 2:** COVID-19 and shipping freight correlation

COVID-19	Correlation	*t*-value	*p*-value
BDI	0.834	32.727^***^	0.000
BDTI	− 0.500	12.491^***^	0.000
BCTI	− 0.397	9.352^***^	0.000

***Significance level.

For the purpose of this analysis, the entire regression series is split into three types, such as short, medium, and long-time horizons. As a result, the short run is defined as a time horizon of 1 to 64 days, while the medium run is a time horizon from 64 to 246 days. Similarly, the long term is a demonstrated time horizon from 256 days onwards.

## Empirical results

**Fig. 1:**
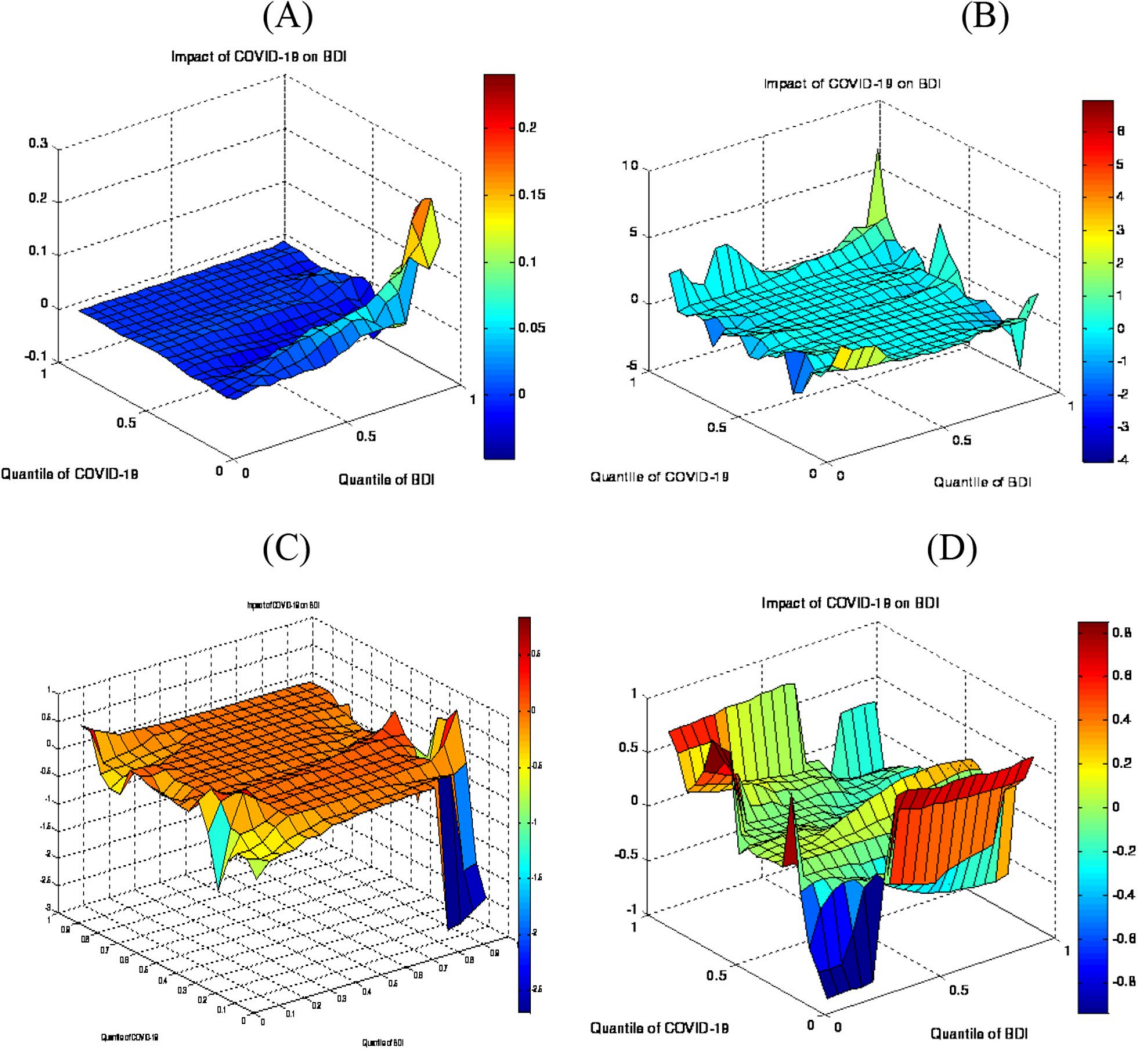
Slope coefficient of QQ estimates. *Note*: the quantiles of COVID-19 are exhibited on the x-axis and BDI on the y-axis

The outcomes of the original variables have been exhibited in Fig. 1A. The τth quantile of the COVID-19 implemented on the $\varphi$ th quantile of BDI is identified by the *z*-axis [15]. The result confirms that the pandemic has a positive effect on BDI in the lower to middle quantiles (0.15–0.65), thus indicating that a pandemic causes a greater rise in BDI.

The decomposed series results that have been extracted in the short, medium and long run have been highlighted in Fig. 1B–D. In the short term, the pandemic positively affects BDI, particularly in the high quantiles (0.80–0.60), which unveils that the instability prompted by COVID-19 induces an increase in BDI in the short run. Meanwhile, the pandemic has affected the BDI in the lower to upper quantiles (0.30–0.90) in the medium run. The unexpected bounce back from the initial lockdown has resulted in increasing shipping demand. COVID-19 has changed consumption and shopping trends and e-commerce is on the rise, while a large part of it is transported by containers [43]. The positive influence has been shown in the lower to upper quantiles (0.20–0.80) in the long run. It shows that the lockdown has resulted in a supply disruption and has squeezed the maritime trade in the initial stages of the pandemic, as reflected in the BDI.

The estimates of the quantile regression tend to permit specific evaluations to be realized for various quantiles of the dependent variable. In this context, “The quantile regression in this case is based on the $\varphi$ *th* quantile of the C OVID-19 on BDI. Also, the parameters of the COVID-19 and BDI are indexed by $\varphi$ and *τ*. Moreover, the QQ method covers more disaggregated material about the COVID-19 and BDI relationship than the quantile regression. The QQ method is heterogeneous across different quantiles. In this regard, the approach is used primarily because of the decomposition characteristics that are used to improve the estimates from the standard quantile regression. Hence, the parameters of quantile regression are indexed by $\varphi$, which is estimated by averaging the QQ parameters along *τ*. The coefficient computes the COVID-19 effect on BDI and is exemplified by $\beta_{1} \varphi$ as follows:

21 $$\beta_{1} \varphi \equiv \overline{{\widehat{{\gamma_{1} }}}} \left( \varphi \right)\frac{1}{s} = \frac{1}{s}\mathop \sum \limits_{\tau } \hat{\gamma }_{1} \left( {\varphi ,\tau } \right)$$

where denote the quantile numbers *τ* = [0.10,0.15,….0.0.90]”.

**Fig. 2:**
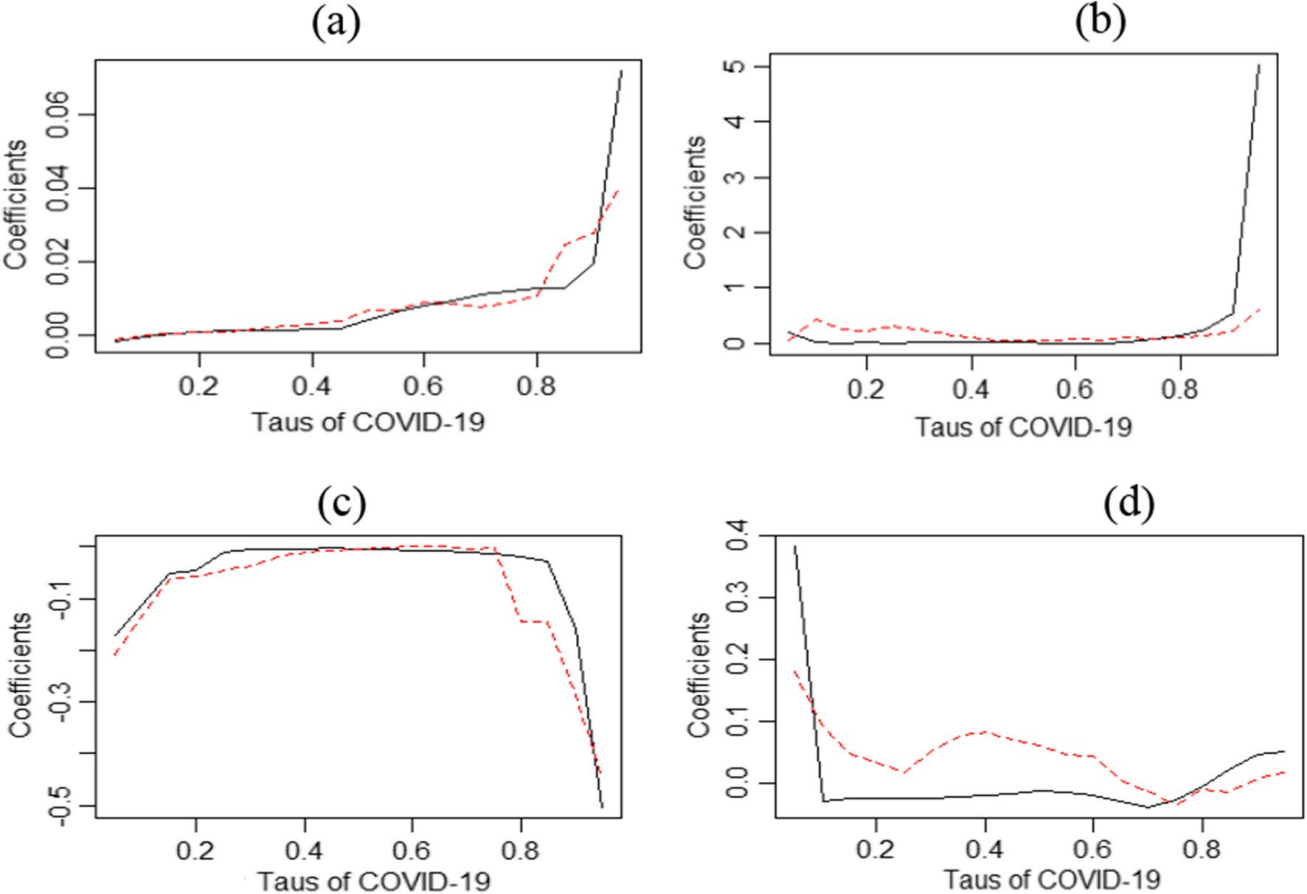
Estimates and regression of quantile

Figure 2a–d explains the estimates and the quantile regression to validate the results that have been achieved. It demonstrates that the pandemic has a positive impact on BDI in all quantiles during the short and long run, while it is negative in the medium run. Moreover, the impact of COVID-19 on BDI is spotted in the upper quantile, thus indicating that the instability produced by COVID-19 has led to the main increase in the freight costs [15]. However, in the long run, BDI is more sensitive to the supply disruptions, and the rising demand from the pandemic has tended to put pressure on the BDI.

**Fig. 3:**
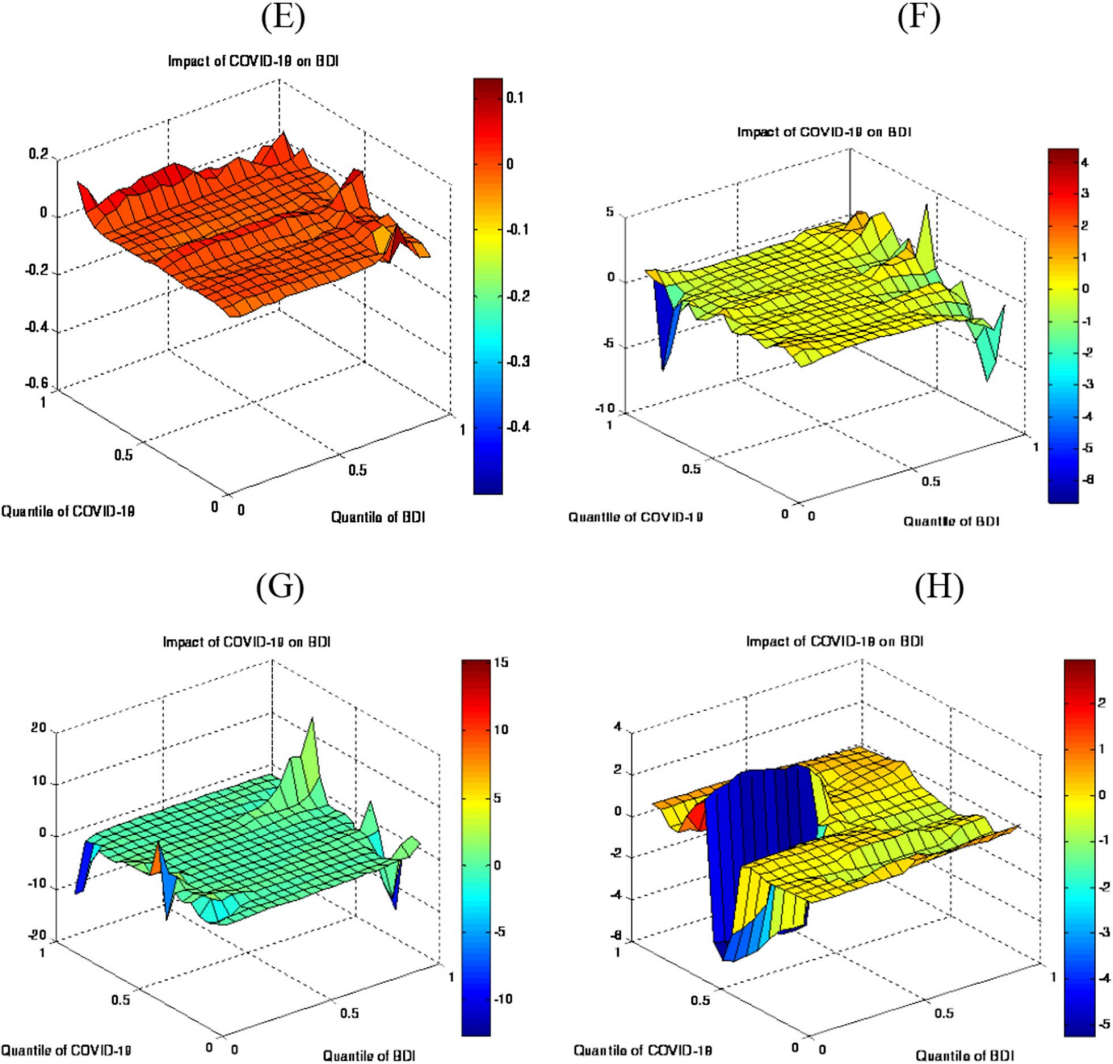
Slope coefficient of QQ estimates

Figure 3E exhibits the COVID-19 impact on BDTI. This explores a significant influence of COVID-19 on the BDTI in the middle to upper (0.55–0.85) quantiles, which reveals that a greater increase in the cost is caused by a higher level of uncertainty due to the pandemic. It also implies that the BDTI shows higher volatility due to the plunge in oil prices.

Figure 3F–H highlights the decomposed data outcomes. It shows that in the short term, COVID-19 negatively influences the BDTI in the upper quantiles (0.55–0.80). It also explores that the unpredictability produced by COVID-19 translates into a rapid decrease in the BDTI. Meanwhile, some countries have not formulated policies to deal with COVID-19, a concept that has a mixed effect on shipping. The pandemic however has had major effects on the industrial production, and the energy usage has decreased dramatically in this regard. Whereas COVID-19 has a distressed BDTI in the midterm, particularly in the upper quantiles (0.75–0.80). It implies that the lockdown has caused the fall of oil demand, thus “turning negative for the first time in history” and as a result, the BDTI experiences a decline. The positive effect is found in the lower to medium quantiles (0.65–0.70), and that too in the long term.

**Fig. 4:**
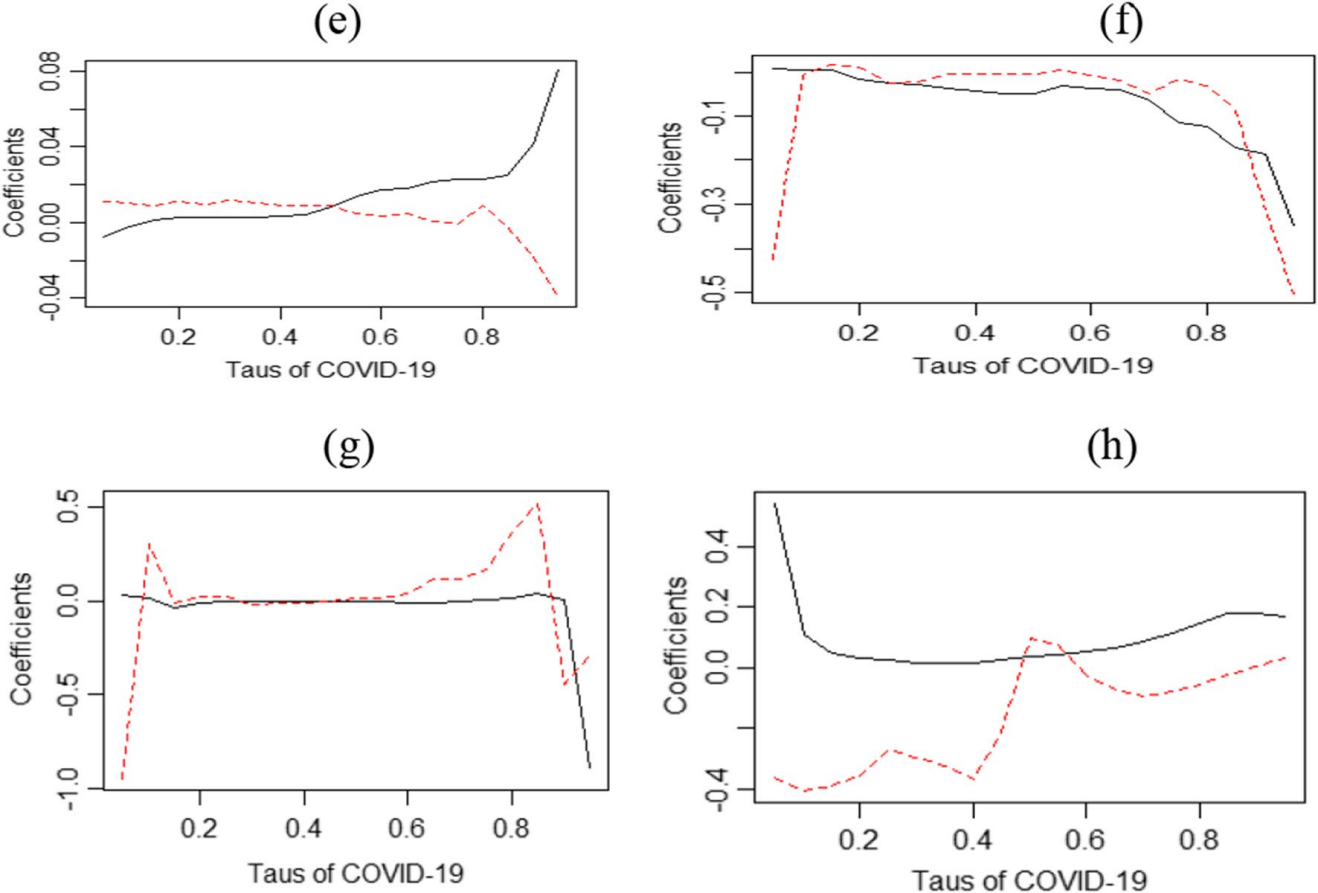
QQ estimates and quantile regression

Figure 4e–h highlights the average QQ regression and estimates to confirm the validity of the result. It establishes that the effect of COVID-19 on BDTI is positive in the overall period that has been considered, while it is negative in the short and medium-terms [15]. Moreover, the results recommend that the impact is more causal in the short- and long-run.

**Fig. 5:**
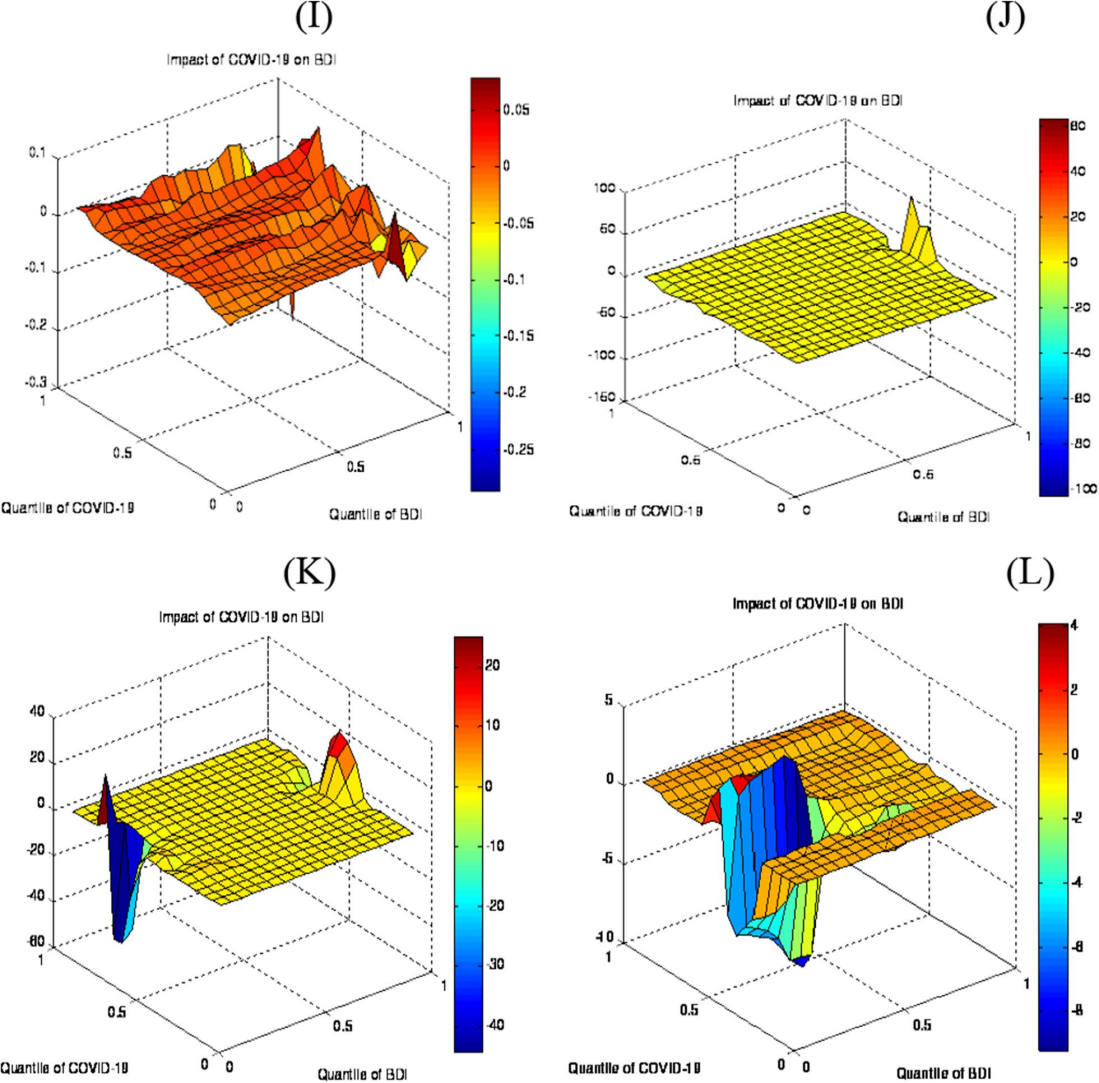
Coefficient and estimates of QQ

Figure 5 (1) exhibits the main series of the COVID-19 impact on BCTI. The result of the main series illustrates a significant positive impact on BCTI in the upper quantile (0.75–0.80). The greater effect has been detected in the higher quantiles, which explore that the pandemic prompts a greater rise in the BCTI.

The decomposed data outcomes have been highlighted in Fig. 5J–L. results show that in the short term, COVID-19 negatively affects BCTI in the medium to upper quantiles (0.60–0.85). This essentially suggests that the unpredictability produced by COVID-19 drives the BCTI to decline rapidly. Meanwhile, COVID-19 has upset the BCTI in the medium to higher quantiles (0.55–0.85), particularly in the medium term. The positive impact has been noticed in the lower to high quantiles (0.30–0.70) in the long term.

**Fig. 6:**
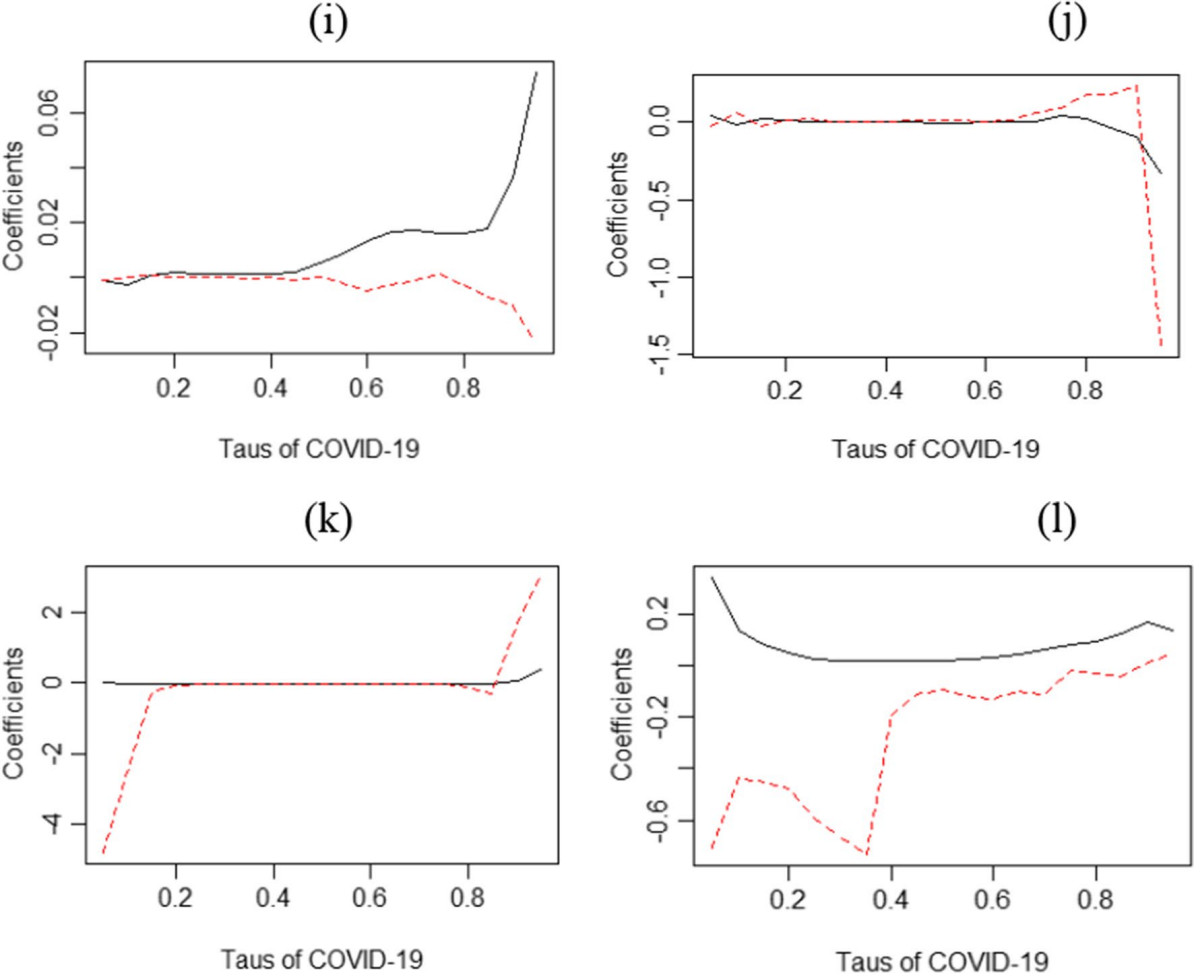
Quantile regression and QQ estimates

Figure 6i–l illustrates the outcomes of different measurements of the QQ regression. It shows a positive impact of COVID-19 on the BCTI during the whole period, and negative across the decomposed period. The outcome recommends that in the long term, the effect is more predictive in nature [15]. However, results show that the BCTI declines rapidly in the first phase of COVID-19, particularly when the demand for energy and raw materials falls in the short run. It can be seen that the uncertainty has increased because of the second wave of the pandemic, and the BCTI decreased in the midterm. The partial recovery of the economy has been observed in the medium term, and the demand for raw materials and energy increased, and the prices stabilized, a concept that pushes freight costs.

Table 3 exhibits QQ estimates for BDI and COVID-19. It shows in panel (1) a significant effect of COVID-19 on BDI in the lower to middle quantiles. The decomposed series results highlighted that the pandemic affects BDI upper quantiles in the short run. However, the pandemic has affected the BDI in the lower to upper quantiles in the medium and long run. The results of the COVID-19 impact on BDTI exhibits in panel (2). The finding shows that COVID-19 has a significant impact on the BDTI in the middle to upper quantiles. Similarly, COVID-19 has significant effects on the BDTI in the upper quantiles in the decomposed series. The results about the COVID-19 impact on BCTI exhibits in panel (3). The result of the main series illustrates a significant positive impact on BCTI in the upper quantile. The decomposed data outcomes show that COVID-19 negatively affects BCTI in the upper quantiles. Meanwhile, COVID-19 negatively affects the BCTI in the medium to higher quantiles in the medium term.

**Table 3:** QQ estimates for shipping freight and COVID-19

Quantile Coefficients	BDI	BDI.D1	BDI.D2	BTC.D3	BDTI	BDTI.D1	BDTI.D2	BDTI.D3	BCTI	BCTI.D1	BCTI.D2	BCTI.D3
1					2					3		
0.05	0.0092	0.0777	− 0.3259	− 0.0921	− 0.0026	0.3109	0.1070	2.2428	− 0.0025	− 2.6937	− 4.8370	− 3.9226
0.10	0.0013	− 0.1597	0.1810	− 0.0451	− 0.0127	0.6349	0.0415	2.5658	0.0062	− 0.2808	− 1.6397	− 4.0189
0.15	0.0021^*^	− 0.0906	0.2664	− 0.0397	− 0.0167	0.3282	0.0245	2.6932	0.006	− 0.1925	− 1.1221	− 4.0154
0.20	− 0.0017	− 0.0909	0.2782	− 0.0592^*^	− 0.0180	0.2312	0.0157	2.9781	0.0065	− 0.1935	− 0.1520	− 3.5659
0.25	− 0.0094	− 0.1094	− 0.003	− 0.1051	− 0.0185	0.1137	0.0159	2.9616	0.0057	− 0.0571	− 0.0905	− 2.2985
0.30	− 0.0046	− 0.0218	0.0017^*^	− 0.1324	− 0.0225	0.2366	− 0.0004	2.8018	0.0058	− 0.3786	− 0.0835	− 1.3751^*^
0.35	− 0.0009	− 0.0213	− 0.0047	− 0.1779	− 0.0216	0.2806	− 0.0111	2.6709	0.0078	− 0.3571	− 0.0712	− 1.4384
0.40	− 0.0006	− 0.0007	0.0038	− 0.2498	− 0.0226	0.2046	− 0.0083	2.4942	0.0182	− 0.4380	− 0.0586	− 0.9207
0.45	0.0012	0.0203	0.0128	− 0.3255	− 0.0163	0.0634	− 0.0076	2.5352	0.0244	− 0.2737	− 0.0559	− 0.8745
0.50	0.0042	− 0.0108	0.0204	− 0.3873	− 0.0164	0.0658	0.0208	2.1545	0.0161	− 0.2135^*^	− 0.0508	− 0.9466
0.55	0.0032	0.0086	0.0445	− 0.4468	− 0.0112^*^	0.0735^*^	0.0357	1.5066	0.0178	− 0.1343^*^	− 0.0307^*^	− 0.7458
0.60	0.0029	0.0523^*^	0.0473	− 0.4899	− 0.0150	0.0560	0.2282	0.1604	0.0206	− 0.0705^*^	− 0.0446^*^	− 0.7036
0.65	0.0019^*^	0.0657^*^	0.0562	− 0.5143	− 0.0172	0.0335	1.0092	0.0858^*^	0.0196	− 0.0313^*^	− 0.0647^*^	0.1103
0.70	− 0.0003	0.1485^*^	0.0461	− 0.5531	− 0.0141	0.0559	1.1728	0.0707^*^	0.0202	0.3816	− 0.0459^*^	− 0.0545^*^
0.75	− 0.0013	0.1407^*^	0.0479	− 0.5471	− 0.0111	0.3730	1.9275^*^	0.0883	0.0246^*^	0.5317	− 0.0833^*^	− 0.2979
0.80	− 0.0002	0.0697^*^	0.0608^*^	− 0.5794^*^	0.0074	0.3955^*^	4.3363^*^	0.0658	0.0261^*^	2.5522	0.1129	− 0.4026
0.85	− 0.0002	− 0.1323	− 0.0510	− 0.5791	− 0.0108^*^	0.6767	8.9613	0.1023	0.0302	3.0445	0.9970	− 0.4338
0.90	− 0.0167	0.2793	− 0.7114	− 0.5897	− 0.0270	1.0279	8.4360	0.1507	0.0395	− 2.2483	1.3084	− 0.4304
0.95	− 0.0004	3.8023	0.5131	− 0.5585	− 0.0313	− 0.5925	15.3024	0.2510	0.0463	− 102.928	17.6509	− 0.3105

Note: * denotes the significance level. D1, D2, and D3 denote the decomposed data for the short, medium and long run, respectively.

## Discussion

Going into a complete lockdown induces the lowest economic activity. That is to say that the raw material demand, amongst other things, also falls because of the closure of the manufacturing industries which, in the case of the COVID-19 pandemic, prompted the reduction in the BDI. These results are similar to the findings of Michail and Melas [27], who found that the pandemic reduced the demand for goods due to restrictions that put forth significant consequences on the BDI. The demand for goods increased as a result of the economic recoveries in China, particularly in the second quarter of 2020, which promoted the shipping demand. However, the shortages of vessels made the trade flow slow and contributed to the increase in the cost [29]. Moreover, the rising demand for goods and meeting the disrupted supply chains were reflected in the highest level of freight cost in the medium run. Meanwhile, maritime trade seemed to have gained momentum, as the governments eased the blockade measures, stimulus plans, and goods hoarded by the companies in response to the new wave of the pandemic. However, the supply could not meet the strong unexpected demand, which then led to the shortages of empty containers, and hence, the highest ever shipping freight.

The widespread closures in the economies around the world, to curb the spread of the virus, have had an adverse impact on the demand for oil tankers. The outcomes are similar to the findings of Khan et al. [14] who found that the pandemic has reduced the demand for goods primarily because of the lockdown which has had significant consequences on the BDTI. The commodity prices, such as oil prices, have declined to a historically lower level, and the transportation and logistics sectors are adversely affected as well, which has shrunk the global economy in the first quarter of 2020 [46]. Furthermore, some countries have had to enforce varying periods of quarantine which has caused delays in sailing and re-routing the commodities via sea. It must be noted that the lowest level was reached in the second quarter of 2020, and in fact remained low in the year 2020. However, the BDTI increased due to the rising oil prices in the first quarter of 2021, indicating the growing demand for floating storage. In this context, the contango state of the oil market made storing oil for future profitable sales possible, which has reduced the availability of vessels for transport; a phenomenon that has exerted pressure on the freight.

The container segment of the shipping industry was struggling before COVID-19 due to lower demand, which has reflected in low container freight costs. Meanwhile, the container segment also experienced impediments because of production halts and trade, and the BCTI touched an all-time low level in the short run. However, the BCTI experienced recovery in the medium run, primarily because the demand for oil-related products increased and there was an economic recovery in some of the affected countries. Moreover, the oil-related products demand increased due to the recovery in Asian countries. In addition to this, the BCTI dropped quickly in July 2020 due to the second wave of COVID-19, lower economic activity, energy prices and uncertainty, which then translated into a deteriorated BCTI. The economic outlook was mixed in the medium term, and the activities returned to normal slowly, while the demand has still been rather uncertain. The iron ore shipment is below average, and the energy prices have declined because of the uncertainty which is reflected in the low BCTI. Similarly, the oil prices reached the highest level in August 2021, combined with a reduction in the supply from the OPEC + members. Moreover, the ease of U.S. sanctions on Iran may have caused a shift in the production locations, which can potentially increase the demand for tankers.

In a nutshell, the response of freight costs has been more responsive to the COVID19 pandemic in the upper quantiles. Moreover, COVID-19 has had a negative effect on the freights in the medium to high quantiles, particularly in the short and medium run. However, the positive impact has also been recognized as appearing in the long term in the high quantiles. The findings support the theoretical model that is taken into consideration, which states that COVID-19 and shipping freights are closely related to one another.

## Conclusion

The study considers the influence of COVID-19 on the shipping freight by employing the QQ approach. The outcome suggests that the pandemic has severely impacted the shipping freight costs, primarily because of the lower demand for the relevant raw materials, energy, unavailability of the vessels, increase in the distances of voyages and logistical inefficiencies. The spread of COVID-19 has increased shipping freight costs in the medium to high quantiles, which suggests that COVID-19 has caused higher level of unpredictability, which ultimately spikes the cost. Moreover, BDI is regularly affected by COVID-19 in the high quantiles, thus showing that higher uncertainty causes a greater increase in the BDI. It also concludes that the lockdown drives a quick fall in the oil demand and raw materials, which is replicated in the BDI. However, BDI has been more responsive to the supply disruption and rising demand, as shaped by COVID-19 in the long term, which has therefore put immense pressure on the BDI. Similarly, in the medium to high quantiles, the pandemic has negatively affected BDTI in the short and the medium run. Whereas a positive impact has been spotted in the medium to high quantiles in the long term. Furthermore, COVID-19 has negatively affected the BCTI in the medium to high quantiles. These outcomes are in line with the theoretical model, which essentially states that COVID-19 and shipping freights are closely correlated to each other.

By comparing the results with the extant literature, it can affirm that the global shipping has been severely affected through sea routes, and by carrying commodities to other countries [11, 17, 19]. Moreover, studies by Arifin [1]; Yazir et al. [46]; Michail and Melas [26]; Menhat et al. [25]; Millefiori et al. [28] show that the pandemic has squeezed the demand for goods, which has ultimately led to negative effects on the international freight costs. Meanwhile, the findings explain that the pandemic has declined demand for raw materials, has caused shortages of vessels, increased distance, and has led to logistical inefficiencies, which reflects in these high freight costs. Furthermore, Michail and Melas [26]; Notteboom et al. [31] and Oyenuga [32] observed that the spread of the pandemic has had a negative impact on the freight cost. These outcomes of the preceding literature are supported by the results showing that the pandemic negatively affected the freight cost in the upper quantiles.

This study offers the following policy recommendations. First, the finding suggests that COVID-19 has had a considerable effect on the shipping freight costs in the long run. Therefore, practical guidelines are requisite to completely understand the extent of the COVID-19 influence on the shipping freight. The outcomes may offer to determine the magnitude of the COVID-19 pandemic and help devise relevant policies to mitigate similar situations in the future. Second, BDI has been observed to be more responsive during the COVID-19 spread in the entire period, which is evident from the lowest and highest level during the pandemic. Therefore, logistics related managers need to pay special attention towards detecting such sudden changes in freight rate dynamics and following such regulations is critical. Third, the precarious situation of shipping in the pre-pandemic period was aggravated by the pandemic. Hence, solving the issue of inefficiencies, vessel imbalance, structural transformation, price war and geopolitical tensions could potentially help to minimize the magnitude of the future crisis. Moreover, the global economy has a derivative effect on the industry, and uncertainty can be disastrous to commodity prices and inflation. As a result, examining the influence of COVID-19 on shipping costs gives a wealth of information that can assist in the mitigation of the risks of sudden changes in freight prices. The recent fluctuation in the shipping freight due to the COVID-19 has attracted great attention to the study of the international shipping market. This research may be expanded by looking at the influence of COVID-19 on shipping freights in the face of fluctuating oil prices. It will evaluate the correlation in the context of the energy market, as oil prices can be a major contributor towards shipping freight volatility. Furthermore, using the wavelet quantile, the influence of COVID-19 on shipping freight may be investigated while using oil price as a control variable.

## Data Availability

Data can be provided at a request.
